# Prognostic analysis and beneficiary identification of adjuvant external beam radiotherapy for stage pT4b sigmoid colon cancer

**DOI:** 10.1038/s41598-021-91172-9

**Published:** 2021-06-03

**Authors:** Yaobin Lin, Lei Wang, Lingdong Shao, Xueqing Zhang, Huaqin Lin, Youjia Wang, Junxin Wu

**Affiliations:** Department of Radiation Oncology, Fujian Medical University Cancer Hospital, Fujian Cancer Hospital, 420 Fuma Rd, Jin’an District, Fuzhou, 350014 China

**Keywords:** Medical research, Oncology

## Abstract

The clinical efficacy of adjuvant radiotherapy in sigmoid colon cancer remains questioned. To evaluate the clinical efficacy of adjuvant external beam radiotherapy (EBRT) for patients with pathologic stage T4b sigmoid colon cancer. Patients with stage pT4b sigmoid colon cancer receiving adjuvant EBRT or not followed by surgery between 2004 and 2016 were extracted from the Surveillance, Epidemiology, and End Results database. Analysis of overall survival (OS) was performed using Kaplan–Meier curves and prognostic factors were identified using Cox proportional hazards regression models with 95% confidence intervals within the entire cohort. A risk-stratification system was then developed based on the β regression coefficient. Among 2073 patients, 284 (13.7%) underwent adjuvant EBRT. The median OS in the group receiving adjuvant EBRT was significantly longer than that in the non-radiotherapy group (*p* < 0.001). Age, serum carcinoembryonic antigen (CEA) level, perineural invasion, lymph node dissection (LND) number, and adjuvant EBRT were independent factors associated with OS. A risk‐stratification system was generated, which showed that low‐risk patients had a higher 5-year survival rate than high-risk patients (75.6% vs. 42.3%, *p* < 0.001). Adjuvant EBRT significantly prolonged the 5-year survival rate of high-risk patients (62.6% vs. 38.3%, *p* = 0.009) but showed no survival benefit among low‐risk patients (87.7% vs. 73.2%, *p* = 0.100). Our risk‐stratification model comprising age, serum CEA, perineural invasion, and LND number predicted the outcomes of patients with stage pT4b sigmoid colon cancer based on which subgroup of high-risk patients should receive adjuvant EBRT.

## Introduction

While the incidence of colon cancer is decreasing steadily worldwide, over 100,000 newly diagnosed colon cancer patients are reported annually^1^. Radical surgery followed by adjuvant chemotherapy is still the preferred curative treatment for locally advanced colon cancer; however, the prognosis remains unsatisfactory, with a 5-year overall survival (OS) rate of 52–64%^2^. Although distant metastasis is decreasing with the development of modern systemic therapy in recent decades, the reported morbidity of local recurrence ranges from 10 to 40%^3–6^, underscoring the role of adjuvant radiotherapy. However, the clinical efficacy of adjuvant radiotherapy, as well as its safety and feasibility, have long been questioned^7,8^.

The radical resection of locally advanced sigmoid cancer is sometimes much more difficult mainly due to anatomical features^9–11^, with a reported proportion of R1/R2 of 15–35%^5,8,12,13^. The incidence of postoperative recurrence for left colon cancer is higher than that of the right^14,15^. However, the sigmoid colon has a relatively fixed location compared with other sites of the colon, which makes adjuvant external beam radiotherapy (EBRT) for sigmoid colon cancer much more acceptable^16,17^. The current study explored the potential role of adjuvant radiotherapy for patients with pathological T4b (T4b: tumor directly invades or adheres to adjacent organs or structures) sigmoid colon cancer in the Surveillance, Epidemiology, and End Results (SEER) database and then established an easily performed model to identify selected patients expected to show more benefits from adjuvant radiotherapy.

## Methods

### Study design and ethics statement

This retrospective study analyzed data from the publicly accessible SEER database. Before the study, we obtained an official permit for the research purpose (ID: 12284-Nov2019). Informed consent or ethical approval was not required for this study.

### Patient selection and data extraction

**Figure 1:**
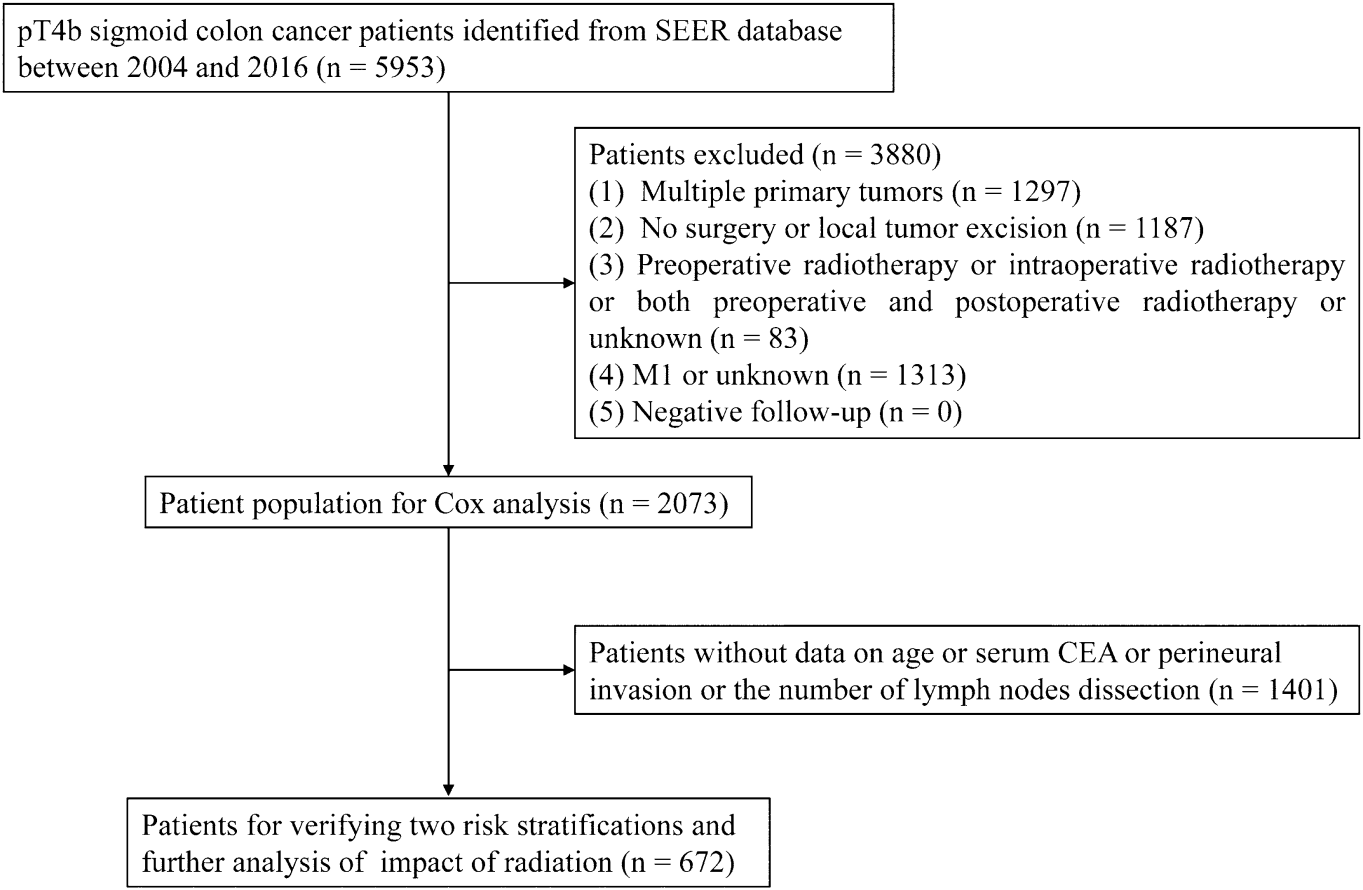
Flow chart of the search protocol and study design.

Cases of stage pT4b sigmoid colon cancer were screened in the SEER database by SEER‐Stat software (SEER*Stat 8.3.8). Cases of sigmoid colon cancer were retrieved based on the International Classification of Diseases for Oncology (ICD-O-3) code; namely, “Site recode ICD-O-3/WHO 2008" (Sigmoid colon). A total of 5953 patients with pT4b sigmoid colon cancer between 2004 and 2016 were initially identified as eligible for this study. Among these, 3880 patients were excluded as follows: (1) 1297 patients had multiple primary tumors; (2) 1187 patients did not undergo surgery or local tumor excision; (3) 83 patients received preoperative radiotherapy or intraoperative radiotherapy or both preoperative and postoperative radiotherapy or an unknown sequence; and (4) 1313 patients were with M1 or unknown. All patients underwent active follow-up. Finally, 2073 patients, including 284 (13.7%) patients receiving radiotherapy and 1789 (86.3%) patients without radiotherapy, were further analyzed (Fig. 1). The T classification was restaged according to the 8th edition of the American Joint Committee on Cancer (AJCC) staging classification system based on the following codes: derived AJCC T, 6th (2004+); derived AJCC, 7th (2010+); and Collaborative Stage (CS) tumor size (2004+)^18^.

The following data were also collected and categorized as follows: insurance (insured, uninsured, or unknown), age at diagnosis (< 50 years, 50–69 years, or ≥ 70 years), sex (male or female), race (white, black, others, or unknown), marital status (married, others, or unknown), differentiation status (well, moderate, poor, undifferentiated, or unknown), tumor size (< 3 cm, 3–4.9 cm, ≥ 5 cm, or unknown)^3,19^, serum carcinoembryonic antigen (CEA) level (elevated, normal, or unknown), perineural invasion (PNI) status (yes, no, or unknown), N classification (N0, N1, N2, or unknown), lymph node dissection (LND) number (< 12, ≥ 12, or unknown)^20^, adjuvant EBRT (yes or no), and survival (months). Data on adjuvant chemotherapy were not extracted in this study, mainly because it was hard to distinguish unknown chemotherapy from no chemotherapy in the SEER database^19,21^, although adjuvant chemotherapy is an important prognostic factor of colon cancer.

### Statistical analyses

The primary endpoint in this study was OS. Kaplan–Meier (K–M) survival curves were determined using log-rank tests. Univariate analysis was conducted on all variables in this study; those with *p* < 0.05 were included in the multivariate Cox regression models to estimate the potential predictors associated with OS.

An easy risk score model was established using the β regression coefficient to predict the prognosis of patients with stage pT4b sigmoid colon cancer, which was assessed by receiver operating characteristic (ROC) curve analysis^22^. The cutoff value affecting OS was determined using the Youden index^22^. K–M survival curves were then used to evaluate the role of adjuvant radiotherapy in the treatment of patients with stage pT4b sigmoid colon cancer stratified according to risk scores.

Statistical tests were conducted using RStudio, including the *Table* *1*, *survminer*, and *survival* packages, or IBM SPSS Statistics for Windows, version 24.0. A *p* value < 0.05 was considered statistically significant.

**Table 1:** Characteristics of patients with stage pT4b sigmoid colon cancer.

Variable	Data, N (%)
**Insurance**	
Insured	1454 (70.1)
Uninsured	117 (5.6)
Unknown	502 (24.2)
**Age (years)**	
< 50	330 (15.9)
50–69	933 (45.0)
≥ 70	810 (39.1)
**Sex**	
Male	988 (47.7)
Female	1085 (52.3)
**Race**	
White	1618 (78.1)
Black	235 (11.3)
Others	209 (10.1)
Unknown	11 (0.5)
**Marital status**	
Married	936 (45.2)
Others	1041 (50.2)
Unknown	96 (4.6)
**Differentiation**	
Well	121 (5.8)
Moderate	1476 (71.2)
Poor	363 (17.5)
Undifferentiated	56 (2.7)
Unknown	57 (2.7)
**Tumor size (cm)**	
< 3	75 (3.6)
3–4.9	391 (18.9)
≥ 5	1512 (72.9)
Unknown	95 (4.6)
**Serum CEA**	
Elevated	724 (34.9)
Normal	514 (24.8)
Unknown	835 (40.3)
**Perineural invasion**	
Yes	186 (9.0)
No	864 (41.7)
Unknown	1023 (49.3)
**N classification**	
N0	1172 (56.5)
N1	535 (25.8)
N2	348 (16.8)
Unknown	18 (0.9)
**Lymph node dissection number**	
< 12	460 (22.2)
≥ 12	1596 (77.0)
Unknown	17 (0.8)
**Radiotherapy**	
Yes	284 (13.7)
No	1789 (86.3)

*N* number, *CEA *carcinoembryonic antigen.

### Ethical approval

For this type of study formal consent is not required.

### Informed consent

As the data used was from SEER dataset (public). Consent to participate could be checked in SEER.

## Results

### Patient characteristics

The clinicopathological characteristics of the 2073 patients eligible for inclusion in this study are summarized in Table 1. The proportions of patients aged ≥ 70 years, tumor size ≥ 5 cm, and LND number ≥ 12 were 39.1%, 72.9%, and 77.0%, respectively; however, only 13.7% of patients received adjuvant EBRT.

### Prognostic factors affecting OS

**Figure 2:**
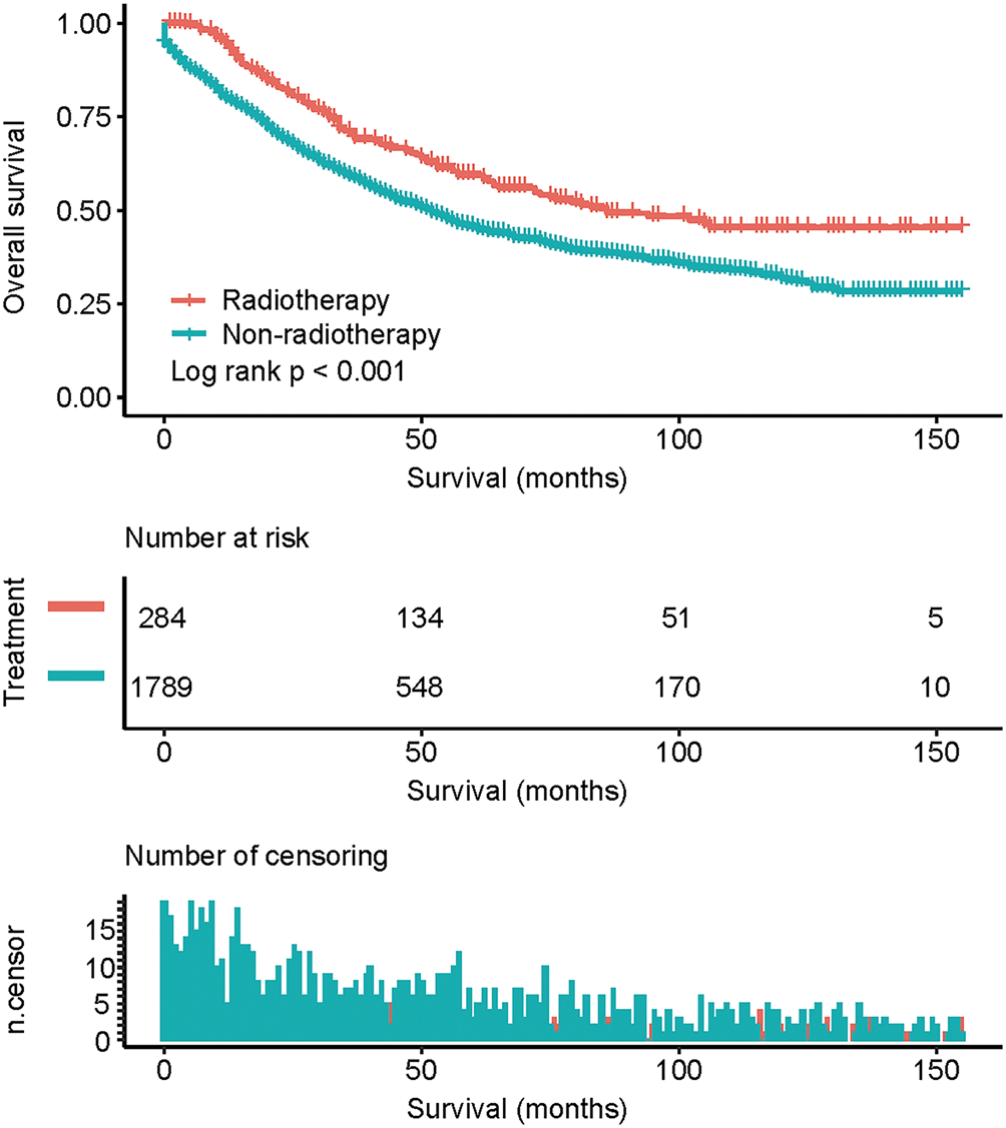
Overall survival (OS) rates for all patients (radiotherapy vs. non-radiotherapy, *P* < 0.001). Pictures show the number of subjects at risk in each group at 50-month increments. Pictures show the number of censoring in each group at 50-month increments.

The median OS times of the adjuvant EBRT and non-radiotherapy cohorts were 84 months and 51 months, respectively, with 1-, 3-, and 5-year survival rates of 94.5% vs. 79.8%, 70.9% vs. 59.8%, and 59.4% vs. 45.5%, respectively (*p* < 0.001, Fig. 2). Univariate analysis showed that age (*p* < 0.001), race (*p* = 0.023), marital status (*p* < 0.001), differentiation (*p* < 0.001), tumor size (*p* = 0.027), serum CEA (*p* < 0.001), PNI (*p* < 0.001), N classification (*p* < 0.001), LND number (*p* < 0.001), and adjuvant radiotherapy (*p* < 0.001) were associated with OS (Table 2). Age ≥ 70 years (*p* < 0.001), elevated serum CEA (*p* = 0.012), PNI (*p* = 0.025), LND number < 12 (*p* < 0.001), and no radiotherapy (*p* = 0.016) were independently associated with worse OS (Table 2).

**Table 2:** Variables associated with overall survival according to the Cox proportional hazards regression model.

Variable	Univariable analysis		Multivariable analysis	
	Hazard ratio (95% CI)	*P* value	Hazard ratio (95% CI)	*P* value
**Insurance**				
Insured	Reference	–		
Uninsured	0.738 (0.539–1.009)	0.057		
**Age (years)**		< 0.001		< 0.001
< 50	Reference	–	Reference	–
50–69	1.063 (0.861–1.312)	0.570	0.984 (0.591–1.639)	0.951
≥ 70	2.530 (2.068–3.096)	< 0.001	3.018 (1.841–4.946)	< 0.001
**Sex**				
Male	Reference	–		
Female	1.078 (0.952–1.221)	0.239		
**Race**		0.023		0.083
White	Reference	–	Reference	–
Black	1.027 (0.848–1.245)	0.784	1.236 (0.798–1.916)	0.342
Others	0.729 (0.578–0.918)	0.007	0.533 (0.277–1.023)	0.058
**Marital status**				
Married	Reference	–	Reference	–
Others	1.302 (1.145–1.480)	< 0.001	1.059 (0.760–1.474)	0.736
**Differentiation**		< 0.001		0.050
Well	Reference	–	Reference	–
Moderate	0.852 (0.658–1.103)	0.224	1.217 (0.531–2.792)	0.642
Poor	1.405 (1.062–1.859)	0.017	1.804 (0.751–4.336)	0.187
Undifferentiated	1.189 (0.757–1.866)	0.452	2.939 (0.917–9.414)	0.070
**Tumor size (cm)**		0.027		0.390
< 3	Reference	–	Reference	–
3–4.9	0.911 (0.651–1.274)	0.584	1.844 (0.631–5.384)	0.263
≥ 5	0.760 (0.555–1.042)	0.088	2.016 (0.721–5.634)	0.181
**Serum CEA**				
Elevated	Reference	–	Reference	–
Normal	0.689 (0.577–0.822)	< 0.001	0.646 (0.458–0.910)	0.012
**Perineural invasion**				
Yes	Reference	–	Reference	–
No	0.568 (0.440–0.735)	< 0.001	0.637 (0.430–0.944)	0.025
**N classification**		< 0.001		0.374
N0	Reference	–	Reference	–
N1	1.250 (1.075–1.452)	0.004	1.227 (0.850–1.771)	0.274
N2	1.664 (1.418–1.953)	< 0.001	1.329 (0.842–2.098)	0.221
**Lymph node dissection number**				
< 12	Reference	–	Reference	–
≥ 12	0.605 (0.528–0.692)	< 0.001	0.398 (0.265–0.600)	< 0.001
**Radiotherapy**				
Yes	Reference	–	Reference	–
No	1.568 (1.294–1.901)	< 0.001	2.015 (1.138–3.567)	0.016

*CI* confidence interval, *CEA *carcinoembryonic antigen.

### Establishment of the risk-stratification model

A risk score model was established according to the β regression coefficient and Exp (B) derived from the Cox model (Table 3). Briefly, age ≥ 70 years was scored as three points, elevated serum CEA as two, PNI as two, and LND number < 12 as three.

**Table 3:** Risk scoring system.

Risk variable	B value	Exp (B)	Risk coefficient	Risk score
**Age (years)**				
< 50	0.000	1.000	1.000	0
50–69	− 0.016	0.984	0.984	0
≥ 70	1.105	3.018	3.018	3
**Serum CEA**				
Elevated	0.000	1.000	1.000	2
Normal	− 0.437	0.646	0.646	0
**Perineural invasion**				
Yes	0.000	1.000	1.000	2
No	− 0.451	0.637	0.637	0
**Lymph node dissection number**				
< 12	0.000	1.000	1.000	3
≥ 12	− 0.907	0.398	0.398	0

*CEA* carcinoembryonic antigen.

**Figure 3:**
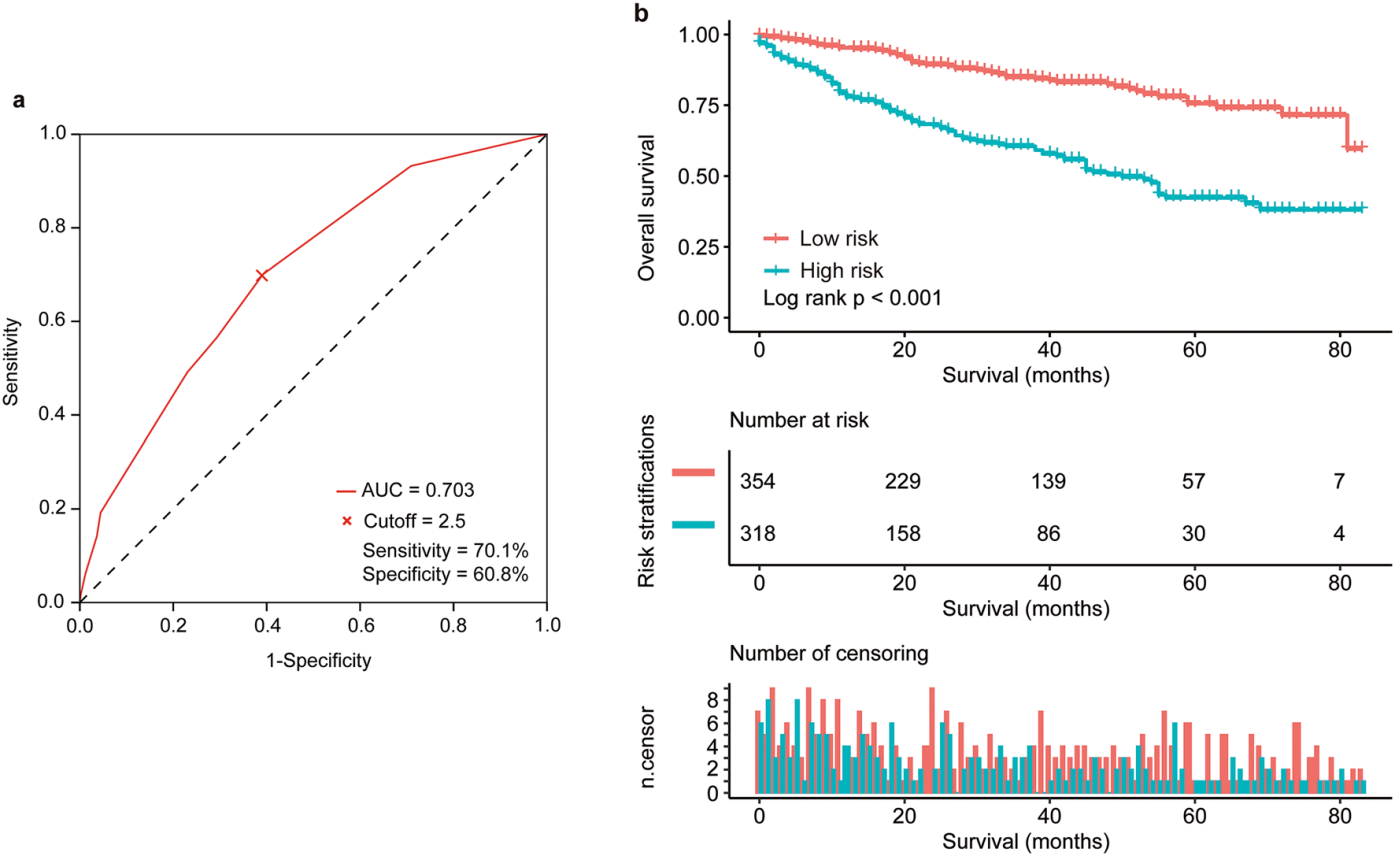
Distribution‐based cutoff optimization for risk score. (**a**) Receiver operating characteristic curve of risk scores; the optimal cutoff was assessed for the event of death. (**b**) Overall survival (OS) rates according to risk stratifications (low risk vs high risk, *P* < 0.001). Pictures show the number of subjects at risk in each group at 20-month increments. Pictures show the number of censoring in each group at 20-month increments.

After excluding patients with missing information for any of the four variables, only 672 (32.4%) patients remained for further analysis. These remaining patients were scored according to the new risk score model. The area under the ROC curve was 0.703, and the optimal cutoff value was 2.5 points (Fig. 3a). According to this cutoff value, 354 (52.7%) patients were classified as having a low risk of poor prognosis (total score < 2.5), while 318 (47.3%) patients had a high risk of poor prognosis (total score ≥ 2.5). The median OS was significantly longer in the low-risk group than in the high-risk group, with 5-year survival rates of 75.6% and 42.3%, respectively (*p* < 0.001, Fig. 3b).

**Figure 4:**
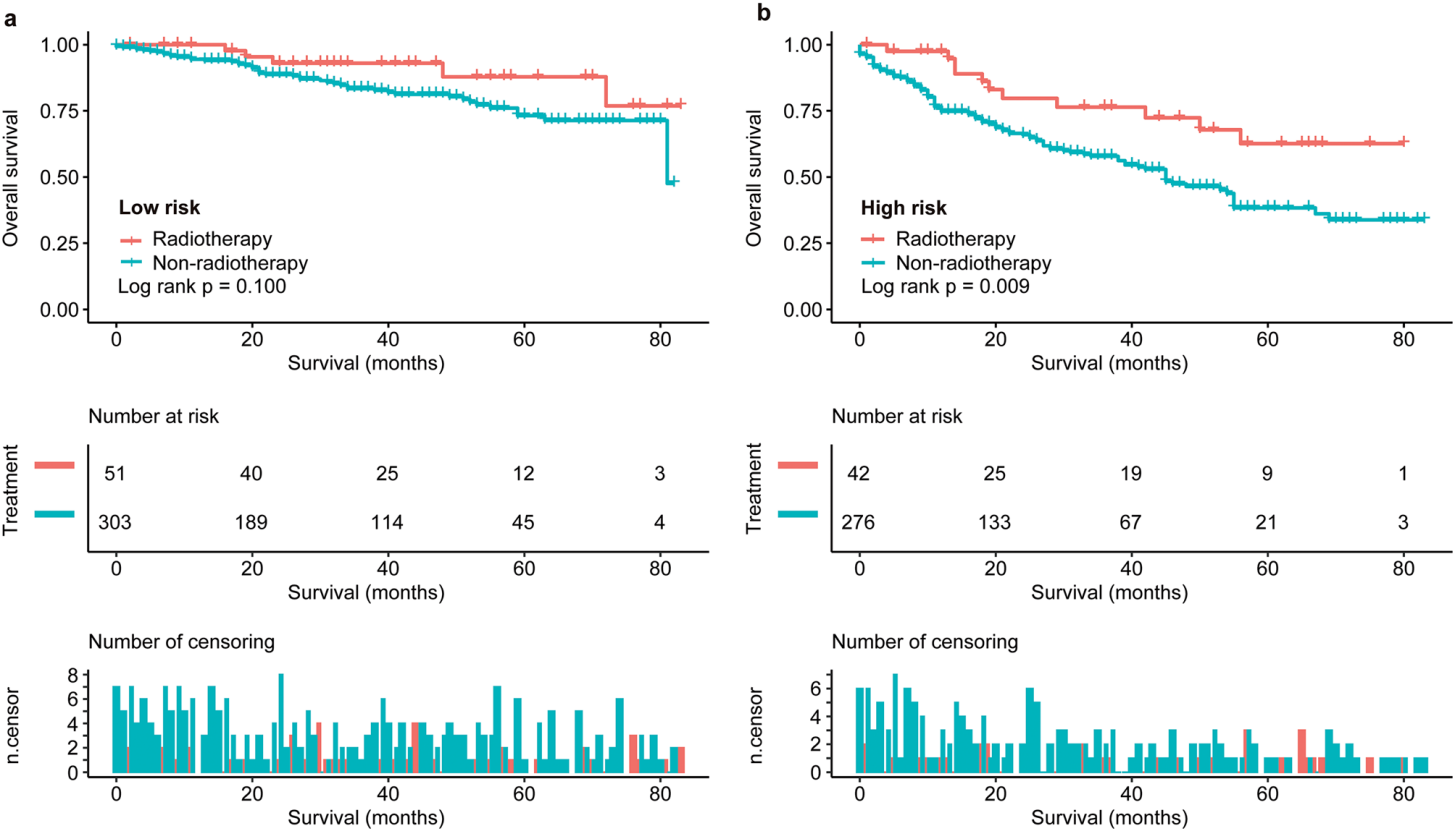
Overall survival (OS) rates according to (**a**) radiotherapy for low‐risk patients (radiotherapy vs. non-radiotherapy, *P* = 0.100) and (**b**) radiotherapy for high‐risk patients (radiotherapy vs. non-radiotherapy, *P* = 0.009). Pictures show the number of subjects at risk in each group at 20-month increments. Pictures show the number of censoring in each group at 20-month increments.

Furthermore, there was no significant difference in the median OS between low-risk patients with and without adjuvant radiotherapy (*p* = 0.100, Fig. 4a). Nevertheless, the median OS in high-risk patients receiving adjuvant radiotherapy was significantly longer than that in patients who underwent surgery alone (not reached vs. 44 months, *p* = 0.009, Fig. 4b), with 5-year survival rates of 62.6% vs. 38.8% (Table 4).

**Table 4:** Survival analysis of patients stratified to two different risk groups.

Risk stratification	With radiotherapy		Without radiotherapy		*P* value
	N	5‐year OS (%)	N	5‐year OS (%)	
Low risk group	51	87.70	303	73.20	0.100
High risk group	42	62.60	276	38.80	0.009

*N* number, *OS* overall survival.

## Discussion and conclusions

To the best of our knowledge, this is the first study to establish an easily assessed risk-stratification model to identify a subgroup of patients with stage pT4b sigmoid colon cancer who might benefit from adjuvant EBRT. According to the new risk stratification model, patients with a total score ≥ 2.5 would benefit more from adjuvant EBRT (*p* = 0.009), whereas patients with a total score < 2.5 would not show a survival benefit from adjuvant EBRT (*p* = 0.100).

Adjuvant EBRT is not routinely used in the treatment of colon cancer. Retrospective studies in the 1980s and the 1990s originally reported improved local control (LC) and disease-free survival (DFS) by adjuvant EBRT^12,23,24^; however, these findings were refuted by a subsequent randomized controlled trial (RCT, Intergroup-0130). No differences in DFS or OS were observed between arms of the trial; however, the results in this trial have always been challenging because of the high ineligibility rates and inclusion of T3 patients^7^. Considering that the patterns of treatment failure have changed in the era of systemic therapy, including targeted therapy and immune therapy^25^, the role of adjuvant EBRT has been re-recognized along with the exploration of preoperative neoadjuvant chemoradiation for colon cancer and decreased toxicity associated with radiation. In 2016, a retrospective single-institution study^5^ reported that adjuvant EBRT could enhance LC and DFS (hazard ratio [HR] 0.044, *p* < 0.05; HR 0.145, *p* < 0.05, respectively) of patients with colon cancer, specifically those with T4b and/or residual tumors, findings that were confirmed using external data or national cancer database^8,26^.

Compared with other sites of colon cancer, adjuvant EBRT for sigmoid colon cancer has several advantages. First, the sigmoid colon is in a relatively fixed anatomical location, which facilitates the delineation of the gross target volume^16,17^. Second, the organs at risk of sigmoid colon cancer are generally fewer than those of other sites of colon cancer. Third, the radiation exposure dose limit for the colon is lower than that for the small intestine. In this study, among patients in the crude cohort, 284 (13.7%) patients with stage pT4b sigmoid colon cancer who received postoperative radiotherapy showed prolonged OS compared with that in patients who did not receive postoperative radiotherapy (*p* < 0.001). As is well known, one treatment size does not fit all. We successfully identified a subgroup of high-risk patients with stage pT4b sigmoid colon cancer who could benefit from adjuvant EBRT based on our newly developed risk-stratification model.

Whether age affects LC and DFS remains controversial^3,27,28^; however, aging is an independent risk factor for poor prognosis of colon cancer, with different aging cutoff values^4,8,21,29^. In this study, these cutoff values were 50 and 70 years and the proportion of patients aged ≥ 70 years was as high as 39.1%. Aging was an independent risk factor for OS, with patients aged ≥ 70 years showing a two-fold increase in poor prognosis, indicating that aging patients, specifically those aged ≥ 70 years, urgently required adjuvant treatment. Moreover, pT4b sigmoid colon cancer typically requires extensive colectomy that aging patients cannot generally tolerate; thus, adjuvant EBRT might be an option for aging patients with stage pT4b disease.

CEA is a routine index used for colon cancer diagnosis and surveillance^30,31^; however, it remains controversial whether elevated CEA levels are an independent risk factor for poor prognosis^14,19,32^. In this study, serum CEA was an independent risk factor for OS. Moreover, elevated serum CEA doubled the risk of death in patients with stage pT4b sigmoid colon compared with that in patients with normal serum CEA levels.

Lymph node evaluation is often a critical factor in predicting the prognosis of colon cancer and is also a deciding factor for postoperative treatment^2,33,34^. However, LND number other than lymph node classification was independently associated with OS, as confirmed in this study, although the cutoff values for LND number varied in previous reports^3,35,36^. In this study, the cutoff value of LND number was 12, as recommended by the National Comprehensive Cancer Network (NCCN) guideline; based on this threshold, LND number < 12 increased the risk of death by nearly two-fold.

PNI is an aggressive characteristic of colon cancer, with incidence rates of 15–32%^14,37^. In a retrospective study of 269 patients with colorectal cancer, Liebig et al.^38^ reported that patients with PNI had a four-fold worse 5-year survival compared with that in patients without PNI, a finding confirmed by several other studies^20,35,39^. However, PNI was also reported to be not associated with poor prognosis in cecum adenocarcinoma or colon cancer^3,14^. Nonetheless, a meta-analysis of 58 studies confirmed the association of PNI with both decreased 5-year OS (relative risk [RR] 2.09, 95% confidence interval [95% CI] [1.68; 2.61]) and DFS (RR 2.35, 95% CI [1.66; 3.31])^40^. In our study, PNI increased the risk of worse OS in patients with sigmoid colon cancer compared with the risk in those without PNI.

This study has some limitations. First, this was a retrospective analysis and selection bias was difficult to avoid. Second, data on local recurrence, a critical endpoint of adjuvant EBRT, were unavailable in the SEER database^41^. Third, while recommended by the NCCN guidelines, this study did not analyze adjuvant chemotherapy, which may have weakened our conclusions. Fourth, data on surgical margin status, microsatellite status, and Ki-67% were not obtained from the current SEER database^18,42^; hence, the newly developed risk model did not include these variables. Finally, details on radiotherapy including clinical tumor volume, radiation technique, dose/fraction, and acute/late toxicity were not recorded^43,44^; thus, the feasibility of adjuvant EBRT requires further study.

In conclusion, the results of this study confirmed the survival benefit of postoperative radiotherapy for pT4b sigmoid colon cancer. A risk-stratification model including the easily measured factors of age, serum CEA, PNI, and LND number was then successfully established. In this model, patients with pT4b sigmoid colon cancer with scores ≥ 2.5 were recommended to receive postoperative radiotherapy.

## Data Availability

The datasets used and/or analyzed during the current study are available from the corresponding author on reasonable request.
